# Separation and Characterization of Prostate Cancer Cell Subtype according to Their Motility Using a Multi-Layer CiGiP Culture

**DOI:** 10.3390/mi9120660

**Published:** 2018-12-14

**Authors:** Lin-Xiang Wang, Ying Zhou, Jing-Jing Fu, Zhisong Lu, Ling Yu

**Affiliations:** 1 Key Laboratory of Luminescent and Real-Time Analytical Chemistry (Southwest University), Ministry of Education, Institute for Clean Energy and Advanced Materials, Faculty of Materials and Energy, Southwest University, Chongqing 400715, China; wlx3952@email.swu.edu.cn (L.-X.W.); zy0200@email.swu.edu.cn (Y.Z.); jingjing1991@email.swu.edu.cn (J.-J.F.); zslu@swu.edu.cn (Z.L.); 2 Guangan Changming Research Institute for Advanced Industrial Technology, Guangan 638500, China

**Keywords:** cells-in-gels-in-paper, cancer metastasis, cell motility, cancer stem cell, drug resistance

## Abstract

Cancer cell metastasis has been recognized as one hallmark of malignant tumor progression; thus, measuring the motility of cells, especially tumor cell migration, is important for evaluating the therapeutic effects of anti-tumor drugs. Here, we used a paper-based cell migration platform to separate and isolate cells according to their distinct motility. A multi-layer cells-in-gels-in-paper (CiGiP) stack was assembled. Only a small portion of DU 145 prostate cancer cells seeded in the middle layer could successfully migrate into the top and bottom layers of the stack, showing heterogeneous motility. The cells with distinct migration were isolated for further analysis. Quantitative PCR assay results demonstrated that cells with higher migration potential had increased expression of the ALDH1A1, SRY (sex-determining region Y)-box 2, NANOG, and octamer-binding transcription 4. Increased doxorubicin tolerance was also observed in cells that migrated through the CiGiP layers. In summary, the separation and characterization of prostate cancer cell subtype can be achieved by using the multi-layer CiGiP cell migration platform.

## 1. Introduction

Cell migration is a fundamental cellular function implicated in many biological and pathological processes, such as embryonic morphogenesis, wound repair, and cancer invasion [1,2,3]. Cancer cell metastasis has been recognized as one hallmark of malignant tumor progression [4]. Metastatic relapse or distant progression is one of the most frequent causes of death from cancer, which clearly emphasizes the urgent need to develop strategies for the prevention of metastasis [2,5,6]. Measuring the motility of cells, especially tumor cell migration, is one approach for evaluating the therapeutic effects of anti-tumor drugs [7]. The Boyden chamber and scratch/would healing assay are routinely conducted in biological sectors to evaluate the migration abilities of tumor cells [8,9]. However, it is not practical to use those methods to harvest and separate cells with different motilities. Recently, Cui et al. developed a microfluidic device containing a biocompatible porous membrane and an array of independently controlled microchambers to isolate and collect migrating cancer cell [10]. By using the microfluidic platform, migrating speed and persistence of breast cells, as well as the morphology and cytoskeletal structures of migrating cells were investigated. However, the gene profile which can underline the migration capability have not been directly studied. Characterizing the phenotype or gene profile of subpopulations that show higher motility will help further elucidate the relationship between the molecular characteristics of tumor cells and their functions [11]. 

In 2009, the novel cells-in-gels-in-paper (CiGiP) method was developed by Dr. Whitesides [12]. The merits of this system include its ease of use and the ability to mimic the three-dimensional (3D) biological, chemical, and mechanical properties of native tissues [13,14]. Since its development, the CiGiP method has been modified to investigate cell invasion, proliferation, and differentiation [15,16,17,18]. For instance, a stacked CiGiP was developed to study cancer cell migration under a gradient of oxygen tension [17]. The monotonically decreasing gradient of oxygen through the CiGiP stack can be easily controlled with the aid of a special holder [16]. Moreover, the impact of the oxygen gradient on the sensitivity of lung cancer cells to ionizing radiation has been evaluated with the CiGiP platform [19]. The flexibility in stacking and destacking the paper [20,21] allows the evaluation of the metabolism of cells at different layers. The CiGiP method provides a versatile tool for exploring the areas of fundamental cell biology and the development of novel therapeutics [12,22,23]. 

Similar to running competitions, we hypothesized that a 3D CiGiP platform could be established to model a cell race (migration), in which the migratory response of cells could be measured by peeling apart the stacked layers. To study the characteristics of cells with different motilities, we established CiGiPs for the model cell, DU 145 prostate cancer cell line. The cells that migrated into different paper layers were isolated and putative biomarkers for prostate cancer stem cells (CSCs) were determined by quantitative PCR (qPCR). To the best of our knowledge, this is the first study to separate and isolate cancer cell subtypes according to their motility, and further compare the CSC-related genes expression of cells with different motility. 

## 2. Materials and Methods

### 2.1. Materials and Reagents

Prostate cancer cells DU 145 were obtained from (Chinese Academy of Sciences Cell Bank, Shanghai, China). The cells were maintained in Dulbecco’s TM® Modified Eagle Medium (DMEM, Gibco, Gaithersburg, MD, USA) containing 10% fetal bovine serum (Gibco), penicillin (100 U/mL) and streptomycin (100 μg/mL) at 37 °C in a 5% CO_2_ atmosphere. Whatman® 105 lens paper and Parafilm® M were purchased from Sigma Aldrich (St. Louis, MO, USA). Matrigel was purchased from Corning (Corning, NY, USA). All other chemicals were acquired from Sigma-Aldrich unless otherwise indicated. All solution was prepared with deionized water produced by PURELAB flex system (ELGA, High Wycombe, UK).

### 2.2. Preparation of the Multi-Layer CiGiP Platform

**Figure 1:**
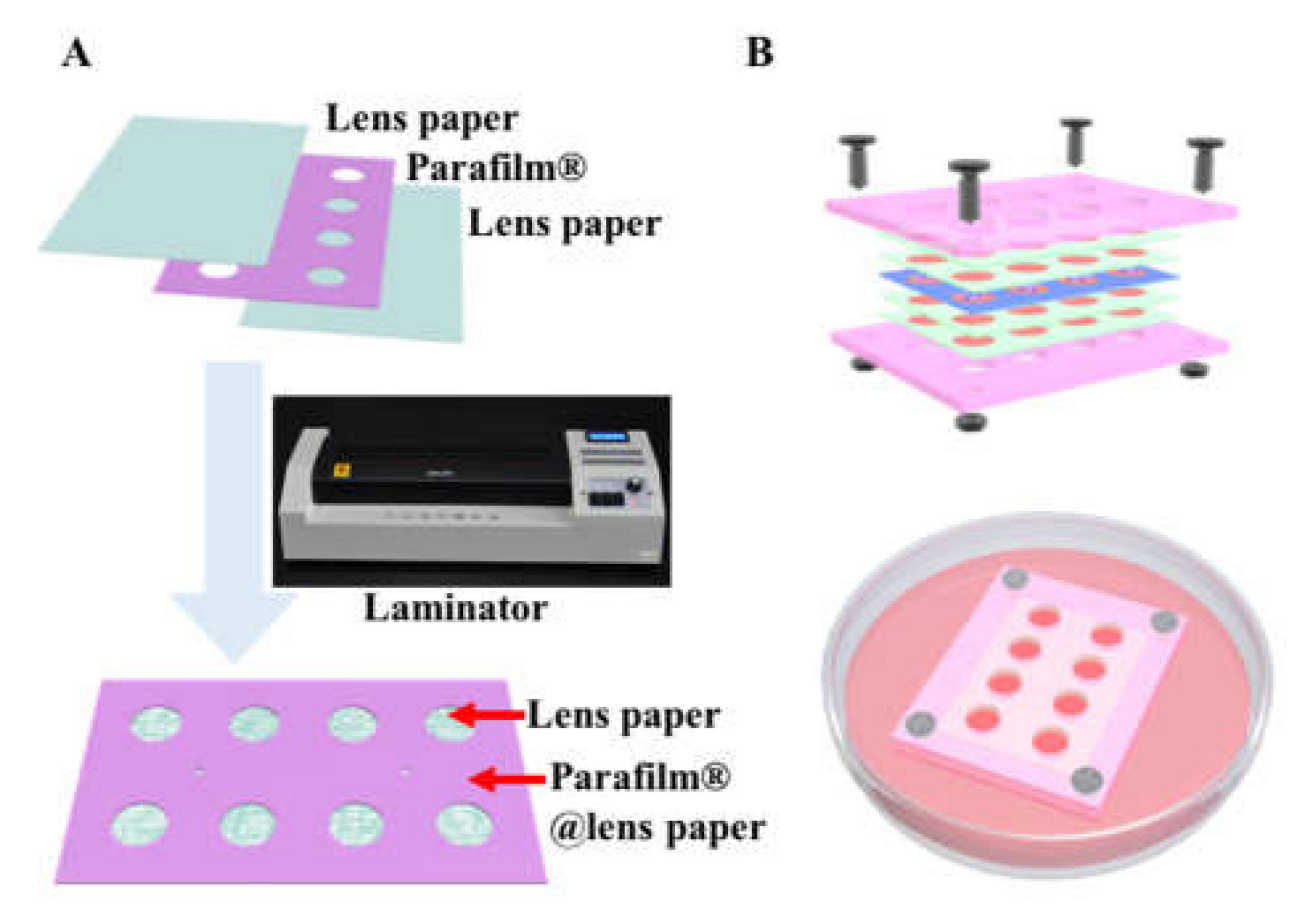
Fabrication of cell-in-gel-in-paper (CiGiP) cell invasion platform. (**A**) Preparing of paper sheet for cell invasion: lens paper–Parafilm® film-lens paper sandwich was sealed with a hot lamination machine to produce single layer of paper sheet; and (**B**) multi-layer paper sheets were assembled and fasten by a chip holder.

*Preparation of the paper sheet*: Lens paper (Whatman® 105, GE Healthcare, Buckinghamshire, UK) was used to prepare the paper scaffold. According to the product information, the thickness of the lens paper is 35 to 40 µm. To form hydrophobic regions on lens paper, commercial wax film Parafilm® (Bemis company. Inc., Neenah, USA) was used. The pattern containing eight holes (diameter of 6 mm) was designed and crafted by a desktop paper crafter. Then a lens paper–Parafilm®-lens paper sandwich structure was assembled and fed into a hot lamination machine (110 °C) to form a single paper stack layer (Figure 1A). The paper stack was sterilized by ethanol washing and ultraviolet light irradiation for 1 h.

*CiGiP-based migration assays*: Cultures of DU145 cells were maintained in tissue culture flasks. Prior to preparing the migration assay, Trypsin-EDTA was used to detach the cells from the tissue culture flask. The Matrigel was thawed at 4 °C. A pre-cooled culture plate and paper sheets were used. All culture plate, patterned lens paper and cell suspension were plated on an ice box during the cell loading process. First, cells were mixed with Matrigel to a final concentration of 12,500 cells/µL. 8 μL cell/Matrigel mixture was spotted into each zone of the seeding layer. The zones in the migration layers were spotted with Matrigel only. Then the paper sheets were incubated at 37 °C for 12 h prior to stacking. The cell-seeded paper sheet was sandwiched between gel-embedded only paper sheets. The multi-layer paper sheets were fastening into a chip holder to ensure the conformal contact of the sheets (Figure 1B). Each multi-layer culture consisted of five layers. The cell-seeded layer was referred to as layer 0. The paper sheets above layer 0 (L0) were layer+1 (L+1) and layer+2 (L+2); the paper sheets below layer 0 were layer-1 (L-1) and layer-2 (L-1). The multi-layer paper sheets were placed in a homemade poly(methyl methacrylate) (PMMA) holder and fastened with screws (Figure 1B). The assembled multi-layer migration stack was placed in petri dishes containing dulbecco’s modified eagle medium (DMEM) and incubated at 37 °C for seven days. 

### 2.3. Characterization of Cell Invasion on Multi-Layer Cultures

To quantify cell migration in CiGiP platforms, multi-layer cultures were de-stacked by disassembling the holder and separating the individual sheets by tweezers. Each sheet was washed with culture medium and phosphate-buffered saline (PBS). To characterize and quantify cell migration, the following experiment was conducted. 

*Fluorescent staining of cells*: The live/dead cell double staining kit, calcein-acetoxymethyl ester (Calcein-AM) and propidium iodide (PI) kit (KeyGEN BioTECH, Nanjing, China), was used for measuring viable and dead cells. In brief, 100 µL Calcein-AM/PI solution was pipetted onto the paper sheets and followed by a 35 min incubation. Then the paper sheets were washed with PBS to remove unstained fluorescence dye. Fluorescence images of the paper-based scaffolds were obtained on a fluorescent microscope (TS100-F, Nikon, Tokyo, Japan). The excitation and emission wavelength for Calcein-AM (staining the living cells) is 495 nm and 515 nm, respectively. While the excitation and emission wavelength for PI (staining of the dead cells) is 535 nm, 617 nm, respectively. 

*Total RNA quantification*: Cells on paper sheets were harvested by Accutase™ cell dissociation reagent (Gibco, Gaithersburg, MD, USA). In brief, 1 mL Accutase™ cell dissociation reagent was added to the paper sheets and incubated at 37 °C for 15 min. Then the paper sheets were washed three times with 0.01 M PBS (pH 7.4). The collected solution was centrifuged at 1000 rpm for 5 min after which the cell pellet was collected. Total RNA was isolated with the TaKaRa MiniBEST Universal RNA Extraction Kit (TaKaRa, Tokyo, Japan) and quantified by DeNovix® DS-11+ spectrophotometry (Gene, Hong Kong, China) at 260 nm.

### 2.4. qPCR to Evaluate Gene Expression in Cells that Invade Different Paper Layers

Cells on each paper sheet were harvested for real time quantitative PCR (RT-qPCR) experiments. Total RNA was reverse transcribed to cDNA using the PrimeScript RT Reagent Kit with gDNA Eraser (Perfect Real Time). PCR primers for OCT4, SRY (sex determining region Y)-box 2 (SOX2), SONG, aldehyde dehydrogenase 1 (ALDH1A1), hypoxia-inducible factor 1-alpha (HIF-1α), and hypoxia-inducible factor 2-alpha (HIF-2α) genes were designed and validated in accordance with the guidelines recommended by the Minimum Information for Publication of Quantitative Real-Time PCR experiments (MIQE) (Table 1). Then qPCR was conducted on the CFX96 Real-Time PCR Detection System (Bio-Rad, Hercules, CA, USA) with SYBR® Premix Ex Taq™ II (Tli RNaseH Plus, Beyotime, Shanghai, China). Glyceraldehyde-3-phosphate dehydrogenase (GAPDH) was used as an internal control. For all reactions, cycling conditions were 95 °C for 2 min followed by 40 cycles of 95 for 15 s and 60 °C for 30 s with a temperature ramp rate of 1.6 °C/s. Amplification profiles were analyzed with QuantStudio 6 and 7 Flex Real-Time PCR System software (MyGo, Gene, Hong Kong, China). The relative expression levels of each gene in cells were normalized to the GAPDH gene using the 2-ΔΔCt method. Three independent experiments were performed.

### 2.5. MTT Assay to Test the Cytotoxicity Effects of Doxorubicin on Cells Migrating Different Paper Sheets

The assembled multi-layer migration stack was placed in petri dishes containing DMEM culture medium and incubated at 37 °C for seven days. Then the multi-layer cultures were de-stacked and each sheet was washed with DMEM culture medium. The paper sheets were separately placed into different wells of the culture plate. Cells in each paper layer were treated with 0.5 μM doxorubicin (topoisomerase inhibitor) for 48 h. Relative cell viability was analyzed. In brief, the paper sheets were washed with PBS and placed in a new well. Fresh medium plus 3-(4,5-dimethylthiazol-2-yl)-2,5-diphenyltetrazolium bromide (MTT) were added to each well and incubated at 37 °C for 4 h, after which dimethyl sulfoxide was added and the wells were incubated with shaking for 15 min. Finally, the absorbance at 570 nm was measured in a microplate reader (ELx800TM, Gene, Hong Kong, China) with a reference wavelength of 630 nm. Cell growth inhibition on each paper layer was calculated using non-drug treated cells as reference samples. For example, growth inhibition on L0 was calculated by: [(A570 of L0 without drug treatment − A570 of L0 with drug treatment)/A570 of L0 without drug treatment] × 100%. Growth inhibition on L+1, L-1, L+2, and L-2 were calculated, respectively. Three independent experiments were performed.

### 2.6. Statistic Analysis 

All experiments were performed three times in triplicate. Data are expressed as the mean ± standard deviation. Results were analyzed with the Student’s t-test using Origin Statistic software (Origin Lab Corporation, Northampton, MA, USA). *p* values less than 0.05 were considered statistically significant.

## 3. Results

### 3.1. Hydrophobic Parafilm® Patterned Lens Paper for Assembling of Multi-Layer Paper-Based Cell Culture Platform

**Figure 2:**
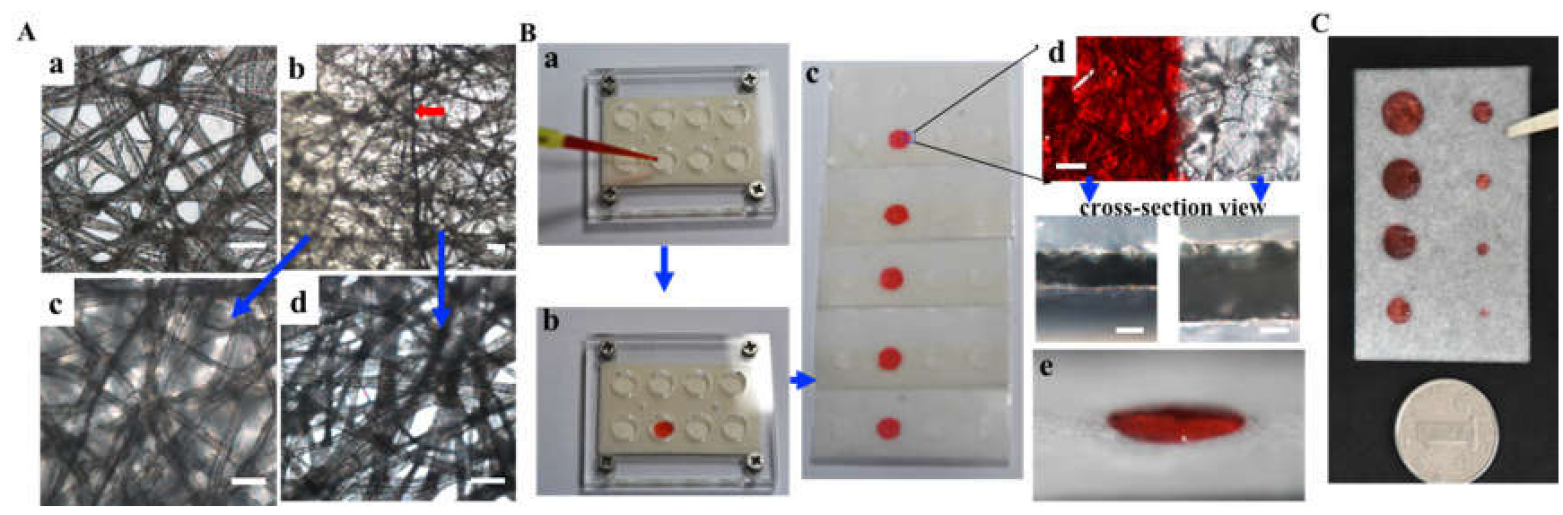
Parafilm® patterned lens paper for assembling of multi-layer paper based cell culture platform. (**A**) Microscopic images of (a) lens paper, (b) Parafilm® impregnated lens paper (red arrow points the edge of Parafilm®), (c) Parafilm®-embedded lens paper, (d) pristine lens paper on Parafilm®-bonded paper, scale bar: 50 µm; (**B**) picture of Parafilm® patterned lens paper: a. assembled multi-layer stack; b. casting solution on cell seeding zone; c. de-stacked multi-layer paper sheet; d. boundary and cross-section of pristine lens paper and Parafilm®-bonded paper, scale bar: 50 µm; e. Matrigel (red) was spotted in hydrophilic region (cell-seeding zone); and (**C**) patterning of hydrophilic region with different size.

In this work, hydrophobic Parafilm® film was utilized to fabricate paper-based substrate for cell culture. Parafilm® is a thermoplastic, self-sealing film that offers excellent barrier protection to the contents of tubes, flasks, culture tubes, etc., in daily laboratory usage. The thermal-sensitive film was sandwiched between two lens papers and then fed into a desktop lamination machine which is normally used for the sealing of photography. The temperature of the hot lamination is 110 °C. During the hot lamination process, the melted Parafilm® can penetrate into the lens paper, forming hydrophobic barriers. Figure 2A-a shows the microscopy image of single-layer pristine lens paper. Pores can be observed between fibers. The average pore size was 37 µm ± 26 µm by randomly measuring 50 pores from the microscopy image. Figure 2A-b–d show the Parafilm®-bonded lens paper. A clear boundary, pointed by red arrow, can be observed between pristine lens paper and Parafilm®-patterned area (Figure 2A-b). Five layers of Parafilm®-bonded paper were stacked and fastened by PMMA holder (Figure 2B-a). Food color dye solution was casted on the cell seeding zone. It was observed that solution only wet the hydrophilic zone of each layer ((Figure 2B-c), indicating that hot-lamination assisted Parafilm® patterning can effectively forming hydrophobic area for stopping fluids (Figure 2B-d). The cross section of the Parafilm®-patterned lens paper was shown in Figure 2B-d. The melted Parafilm® can firmly bond two layer of lens paper. The thickness of the hydrophilic area (pristine lens paper) is around 75 µm ± 14 µm, which similar to the thickness of two single pristine lens paper. While, thickness of the Parafilm®-patterned lens paper is around 135 µm ± 11 µm. Matrigel can be safely held within the cell-seeding region (Figure 2B-e). Previously, wax printing [17] or polyvinyl chloride (PVC) sheet [19] were used for generating hydrophobic barrier for paper-based cell culture. Comparing with those reported methods, Parafilm®-assisted fast patterning does not require a wax printer and high temperature-assisted bonding. By using the cost-effective method, lens paper was patterned with hydrophilic areas in different size. Figure 2C shows that a circle zone with a diameter down to 1 mm can be produced (Figure 2C). In this study, to ensure the uniform contact of multi-layer of Parafilm®-patterned lens paper, while provide sufficient area for cell growth, circle zone with a diameter of 6 mm was design and used. Multi-layers of patterned lens paper can be stacked and fastened with a PMMA frame for cell culture. 

### 3.2. CiGiP Multi-Layer Cultures Assisted Separation and Isolation of Cells According to Their Motility

**Figure 3:**
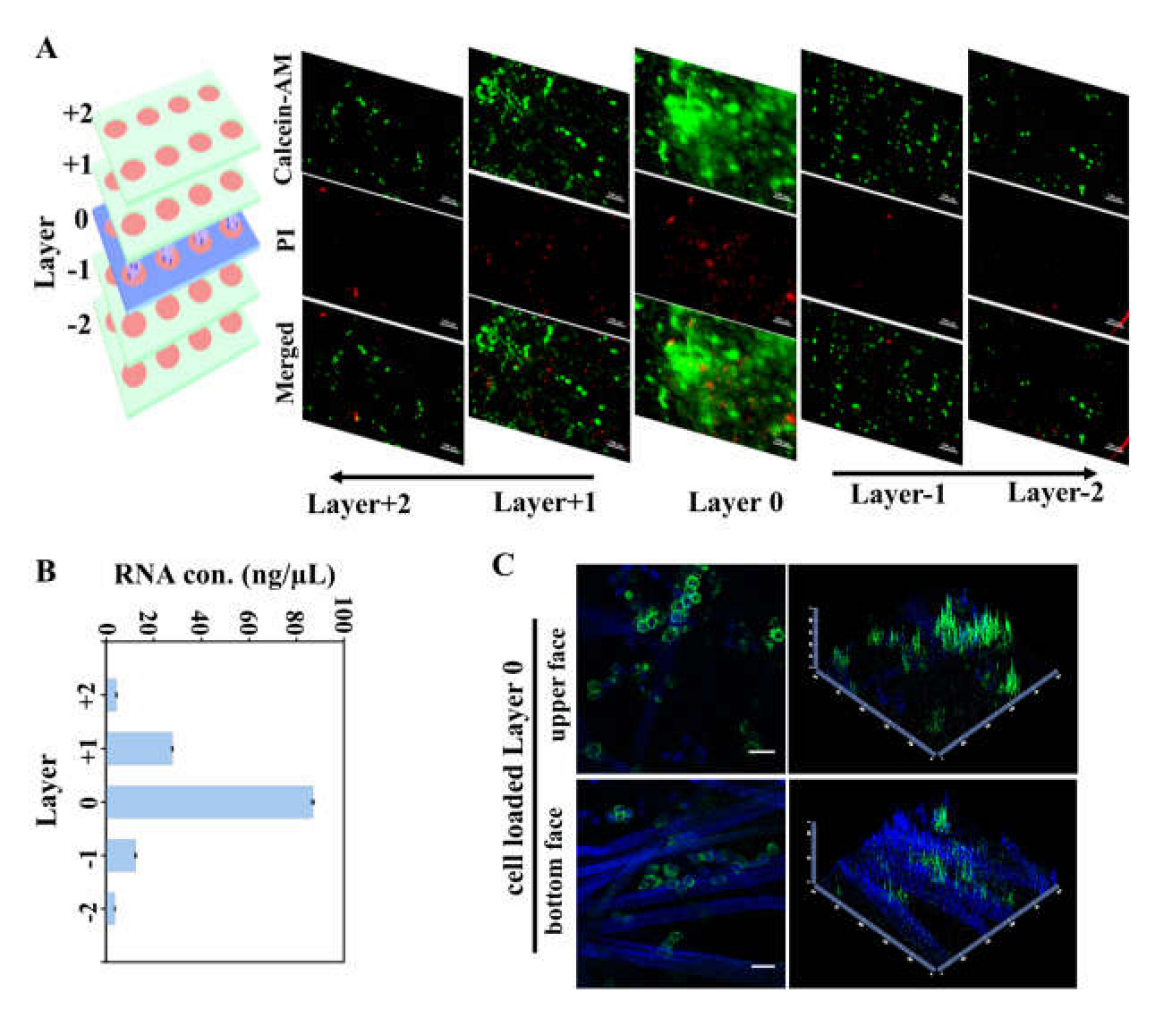
Separation and isolation of cells according to their motility. (**A**) Fluorescent image of cells migrated into different layers of the CiGiP cell migration platform. Prostate cancer DU 145 cells were loaded at the middle layer (Layer 0) of the invasion platform. After seven days culturing, the CiGiP platform was de-stacked. Cells at different layers were stained with Calcein-AM/PI kit. Calcein-AM stains live cell, while PI stains dead cells. Scale bar: 100 µm. (**B**) RNA concentration isolated from cells invaded into different layers of the CiGiP cell invasion platform. (**C**) Confocal microscopy image of cell seeding layer. Eight microliters of cell/Matrigel mixture was spotted into each zone of the seeding layer. The paper sheet was incubated at 37 °C for 12 h. Then the cells on paper sheet was stained with Calcein-AM. The upper face and bottom face of the paper sheet were examined under confocal microscopy (LSM800, Zeiss, Munich, Germany). The 2D images (left panel) were converted into 2.5-D images (right panel) by confocal software Zen. Scale bar: 50 µm.

Cell migration assay was conducted with the stacking CiGiP multi-layer cultures. As illustrated in Figure 1B and Figure 2B, the chip holder used in this study allowed diffusion of the culture medium into the CiGiP structures from both the top and bottom sides, minimizing the nutrition difference between the upper and bottom paper sheets. The cell-seeded paper sheet (L0) was sandwiched in the middle layer of the CiGiP platform, and was the start point of the cell migration. The stacked multi-layer cultures mimic the bulk movement of cells in a tissue-like environment. The multi-layer CiGiP platform was cultured for seven days, and then de-stacking of the paper scaffolds was performed. First, we stained cells on different layers by Calcein-AM/PI kit, which can simultaneously detect live and dead cells. Highly-packed live cells (green) were observed in L0 (Figure 3A). At the same time, dead cells (red) also can be observed after seven days stacking-culture. The cell density sharply decreased in layers above and below L0. The cells traveled into top and bottom layers were visualized by live cell dye Calcein-AM. Since the thickness of the Parafilm®-patterned paper sheet was about 75 μm, the distance the cells travelled within the multi-layer cultures could be quantified. With an increase in traveling distance, the number of cells sharply decreased, indicating heterogeneous motility. 

As shown in Figure 3A and previous studies [12,17], cell staining with dyes facilitated the observation and quantification of live cells in the different layers. The paper sheet was photographed by microscopy or using a scanner. Cell distribution was quantified by image analysis. Due to the 3D structure of the gel embedded paper, it would be difficult to preciously quantify the cells buried in the matrix. To address this concern, we isolated cells from the paper sheet, and measured the levels of total genomic RNA to quantify the cells in each layer. The concentration/amount of RNA in each layer was directly measured from cells isolated from each layer. As shown in Figure 3B, cells in L0 had the highest level of total genomic RNA (86.91 ± 0.18 ng/µL). The RNA amount/concentration sharply decreased in cells from the L+1 and L-1 layers, although the RNA levels in cells from the L+1 layer were higher than those in cells from L-1. We further examined the upper and bottom faces of the cell-seeded sheet (L0) by confocal microscopy (LSM 800, Zeiss, Munich, Germany). As shown in Figure 3C, a higher cell density (green) can be observed from the upper surface of the cell seed layer. The difference between the upper and bottom surfaces of the cell seed layer may due to the gradual gelation of the cell-matrix mixture during penetration through the lens paper, although cell seeding was conducted on an ice-box.

Fluorescent staining assisted live cell imaging and RNA quantification in CiGiP constructs both demonstrate that cells have different motilities. The longer distance the cells travel in the same period of time, the higher their motility capability. Thus the phenotype and molecular characteristics of those cells should be investigated to determine the mechanisms underlying their high motility.

### 3.3. Elevated ALDH1A1, SOX2, NANOG, and OCT4 Expression in Cells with Higher Motility

**Figure 4:**
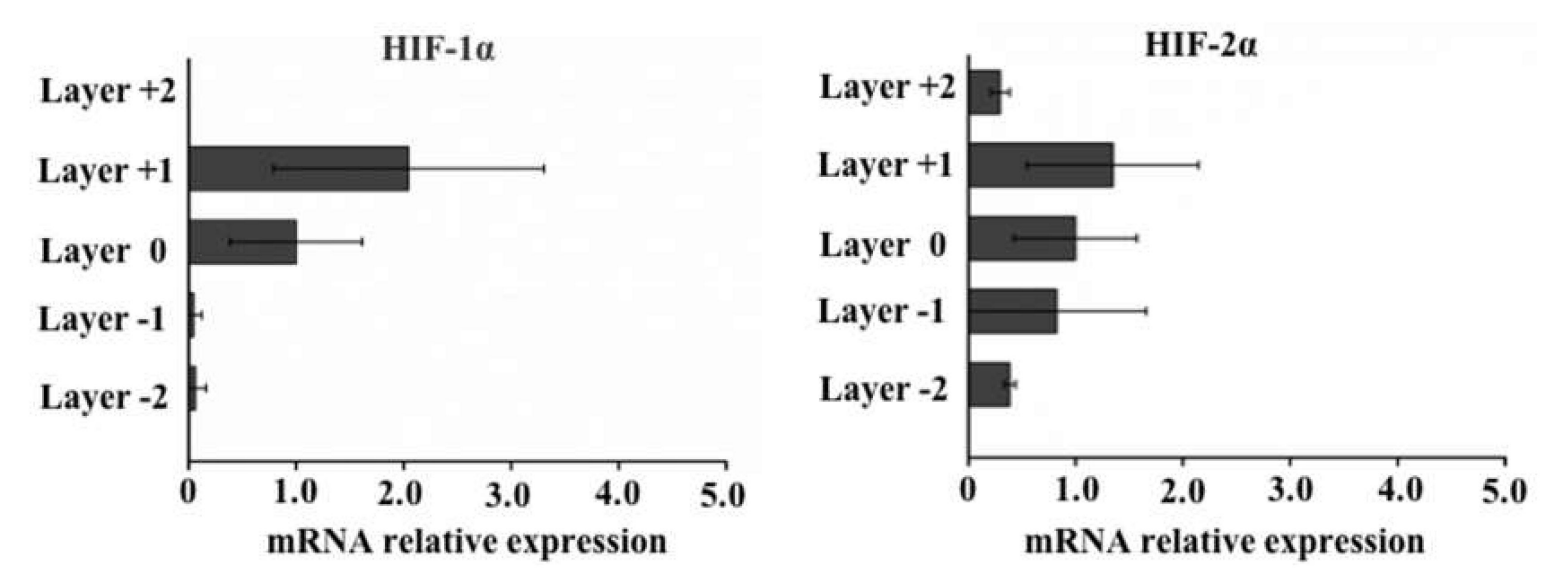
Relative expression hypoxia markers measured by quantitative PCR analysis. Real-time PCR-quantified levels of expression of hypoxia-inducible factor 1-alpha (HIF-1α) and hypoxia-inducible factor 2-alpha (HIF-2α) using 2-∆∆Ct values relative to glyceraldehyde-3-phosphate dehydrogenase (GAPDH) as an internal control. Error bars represent the standard deviation for 24 replicate zones.

By using the CiGiP cell migration platform, subpopulation of cancer cell can be collected from different paper layer. In this study, cell-seeded paper sheet was placed as the middle layer of the paper stack. Culture medium can perfuse from top and bottom side of the hydrophilic region. To exam the effect of stacking caused oxygen and nutrient gradient on cells, the expression of hypoxia-inducible factor 1-alpha (HIF-1α) and hypoxia-inducible factor 2-alpha (HIF-2α) were measured by qPCR. As shown in Figure 4, expression of hypoxia-related HIF-1α and HIF-2α gene is higher at the middle layer than those in outer layer. 

**Figure 5:**
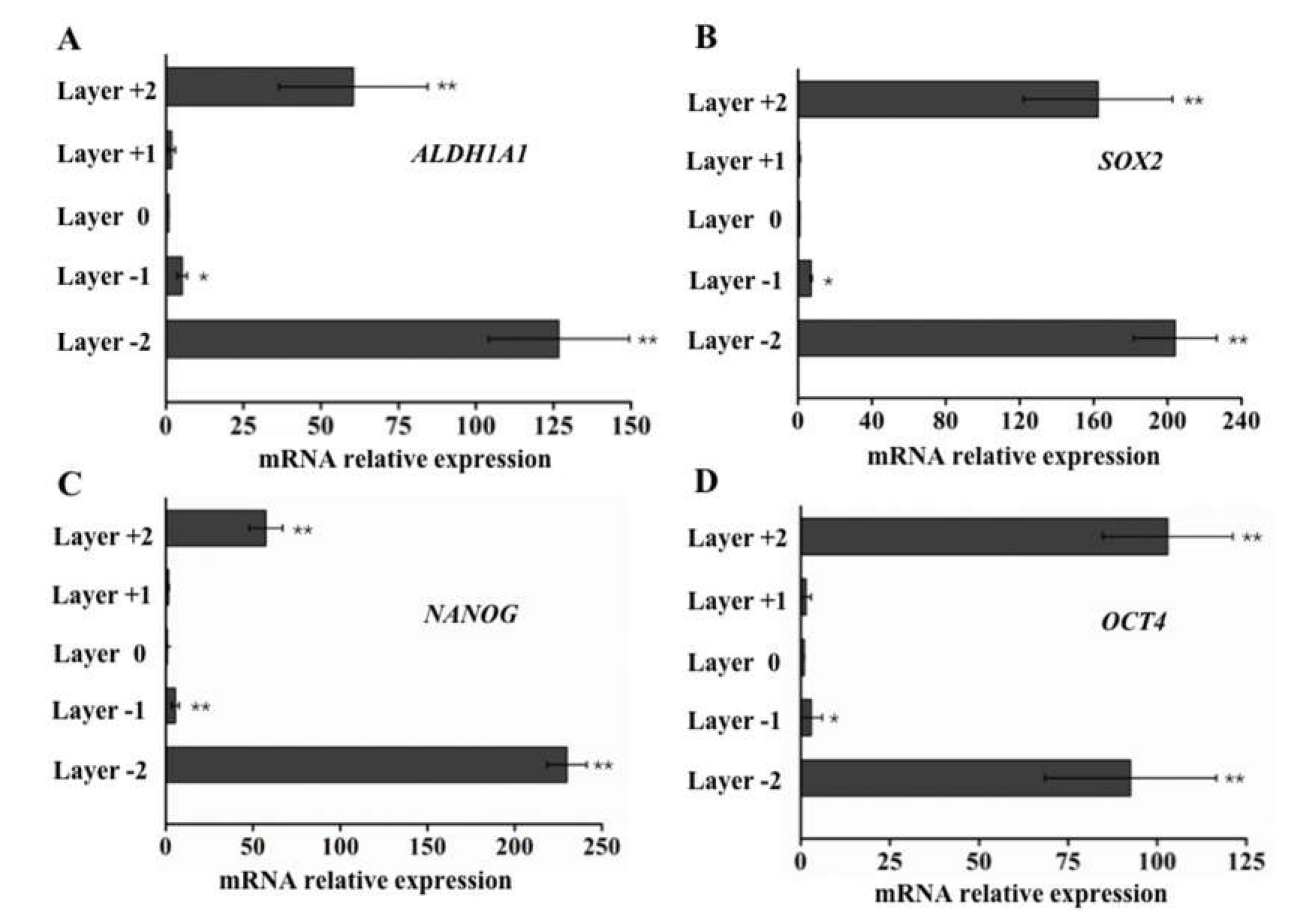
Relative expression of putative CSC-specific markers measured by real-time quantitative PCR analysis. Real-time PCR-quantified levels of expression of ALDH1A1, NANOG, OCT4, and SOX2 transcripts using 2-∆∆Ct values relative to glyceraldehyde-3-phosphate dehydrogenase (GAPDH) as an internal control. Error bars represent the standard deviation for 24 replicate zones, * denotes *p* < 0.05, ** denotes *p* < 0.01.

To further underline the difference of their motility, expression of several cancer stem cell related biomarker were measured. First, aldehyde dehydrogenase 1 (ALDH1A1), a detoxifying enzyme that oxidizes intracellular aldehydes, was studied. ALDH1A1 is also a modulator of cell proliferation and migration [24]. Upregulation of ALDH1A1 has been observed in primary prostate cancer tissues and metastatic lesions. We performed qPCR to evaluate ALDH1A1 expression on cells collected from different layer of paper. Compared with ALDH1A1 levels at the seeding layer, ALDH1A1 levels in cells at L+2 and L-2 were 60.45 ± 23.92 and 126.72 ± 22.71 fold higher than that of L0, respectively (Figure 5A), indicating a significant ALDH1A1 expression increase in cells with higher motility. In addition, the difference in expression at L+2 and L-2 was most likely due to the fact that the upper face of the cells-seeded sheet (L0) had more cells than the bottom face of this layer (Figure 3C). The cells on the upper face were in direct contact with L1, potentially decreasing the traveling distance and causing variation in motility. In our CiGiP-based cell invasion platform, although only a small fraction of cells travelled through the two layers of paper sheets during the seven days of culture, their ALDH1A1 levels were significantly higher than those with less motility. Growing evidence indicates that ALDH1A1 is a putative CSC marker of several types of solid tumor, including prostate cancer [18,25,26]. According to cancer stem cell (CSC) theory, there is a very small fraction of cancer cells that could initiate cancer and propagate metastasis. The heterogeneous expression pattern of ALDH1A1 from cells with stronger motility suggests the relationship between ALDH1A1, cell motility, and potential stemness. Next, embryonic markers, octamer-binding transcription 4 (Oct-4), SRY (sex determining region Y)-box 2 (SOX2), and NANOG [27,28] were studied. The instrumental roles of those molecules in promoting tumor initiation have been identify. Previous studies also found that the embryonic markers SOX2, NANOG, and OCT4 were elevated in CSCs isolated from human prostate cancer tissue, human prostate tumor models, and some prostate cancer cell lines [29,30,31]. To investigate the gene expression levels of these putative prostate CSC biomarkers in cells with distinct motility, qPCR measurement was conducted. The results showed that the expression levels of SOX2, NANOG, and OCT4 in cells from the L+2 layer were 162.40 ± 40.28, 57.39 ± 9.63, and 102.98 ±18.24 fold higher than levels from cells in the L0 layer (Figure 5B–D). The gene expression levels of those putative biomarkers were elevated to 204.15 ± 22.51, 229.95 ± 11.04, and 92.56 ± 24.09 in cells from the L-2 layer. As discussed above, cells that migrate to L-2 have to travel through two layers of Matrigel-impregnated paper sheets, which is probably a longer distance than cells that travel to L+2. Collectively, the elevated ALDH1A1, SOX2, NANOG, and OCT4 expression in cells travelled through different distance would suggest the potential stemness of the small fraction of cells with higher motility. 

### 3.4. Increased Resistance to Doxorubicin of Cells with Higher Motility

**Figure 6:**
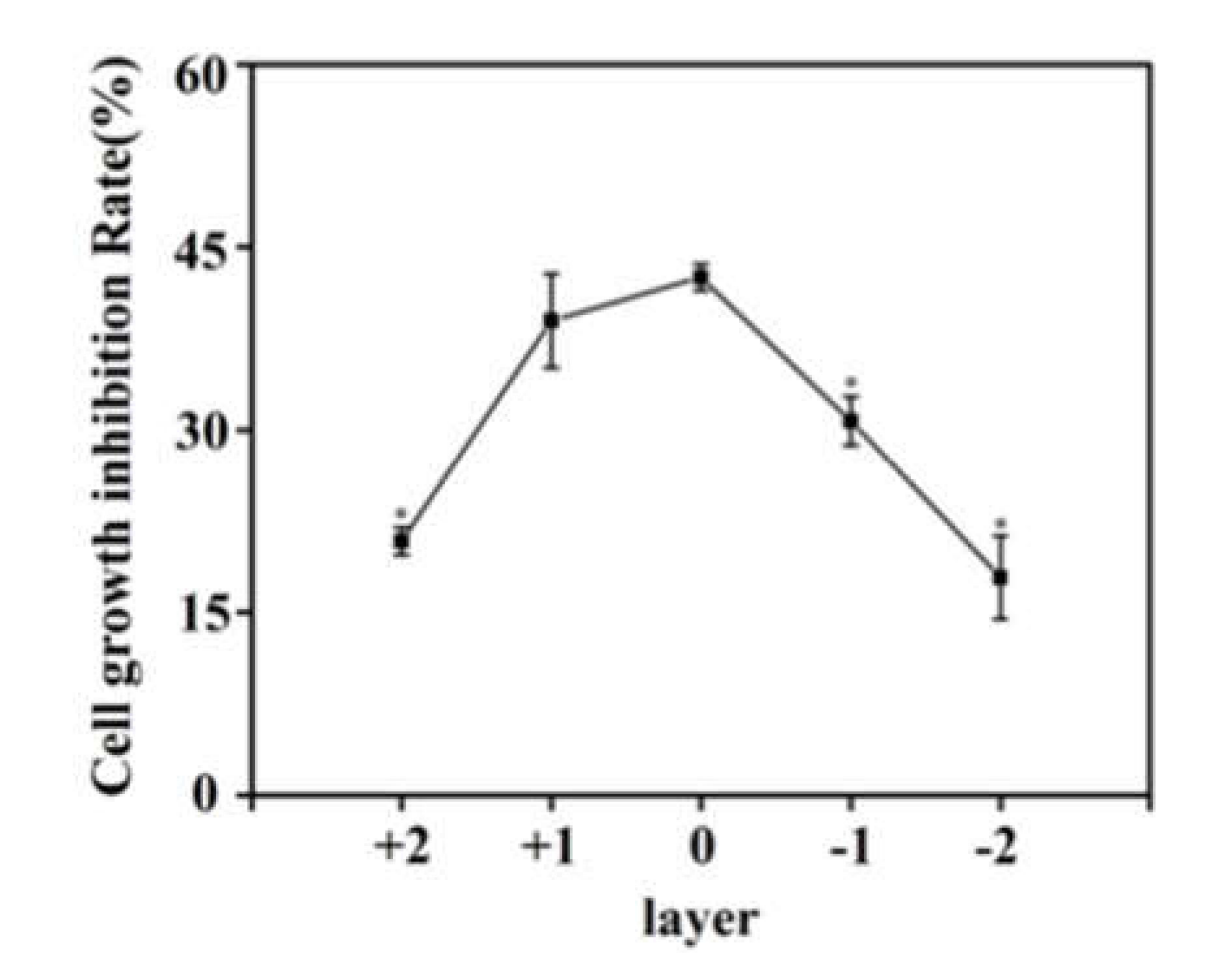
DU145 cells were seeded in layer 0 and the assembled multi-layer migration stack was cultured for seven days. Then each paper sheets were separately placed into culture medium containing 0.5 μM doxorubicin for 48 h. Finally, an MTT assay was conducted with doxorubicin-treated paper sheets. Cell growth inhibition on each paper layer was calculated using non-drug treated cells as reference samples (e.g., growth inhibition on L0 was calculated by: [(A570 of L0 without drug treatment − A570 of L0 with drug treatment)/A570 of L0 without drug treatment] × 100%. Growth inhibition on L+1, L-1, L+2, and L-2 were calculated, respectively. Error bars represent the standard deviation for 24 replicate zones, * denotes *p* < 0.01.

According to the CSC theory, CSCs are more resistant to chemotherapy than differentiated cancer cells [32]. Cells were subjected to treatment with the chemotherapy agent doxorubicin, followed by de-stacking of the CiGiP platform to obtain individual paper sheets. The inhibitory effects of doxorubicin on cell proliferation were evaluated. The cell growth inhibition rate at L0 was 42.50% ± 2.53%. Although only a small fraction of cells successfully invaded to L-2 layer, the cell growth inhibition rate was only 17.85% ± 1.91%, significantly lower than that at L0 (Figure 6). The decreased cell growth inhibition after doxorubicin treatment indicated drug resistance of cells at L-2, which had stronger motility and elevated expression of putative CSC biomarkers. Thus, the CiGiP invasion platform was successfully used in characterizing and separating cells with high invasion and resistance to chemotherapy reagent from bulk cancer cells. Importantly, the heterogeneous gene expression level in these separated cells may help us gain insights into the mechanisms underlying tumor metastasis and drug resistance.

**Scheme 1:**
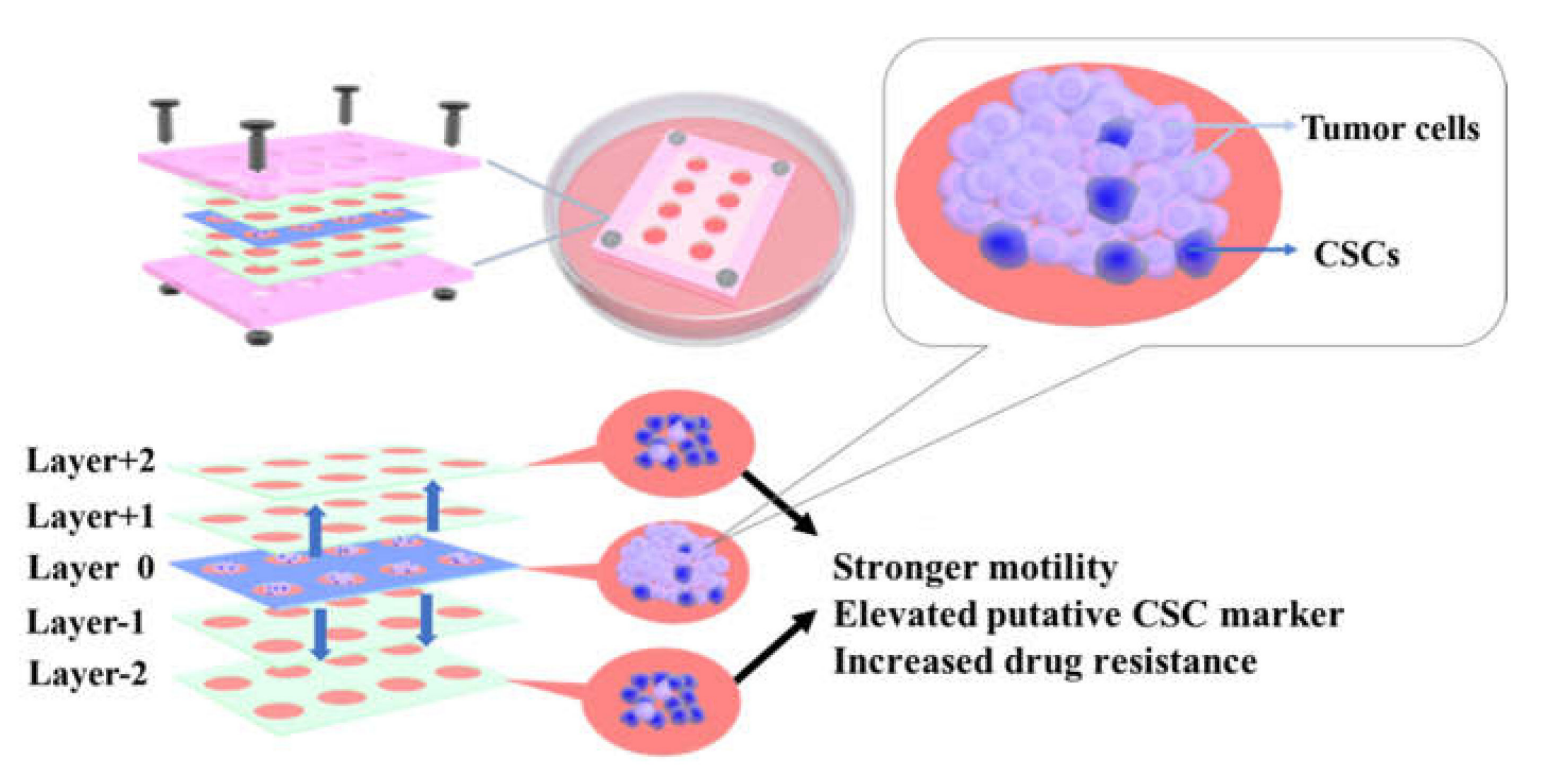
Schematic drawing of the multi-layer CiGiP cell invasion platform mimicking the in vivo tumor metastasis process. Separation and isolation of subtype of tumors according to their motility can be achieved by de-stacking of the multi-layer invasion platform. The elevated expression of the putative cancer stem cell (CSC) biomarker suggests the relationship between stemness and metastasis.

Research studies on cell motility or cell migration are usually conducted using the Boyden chamber transwell or wound healing assay, as these are well-established methods for examining the migration capability of cells, especially after drug challenge [8]. However, in this study, the multi-layer CiGiP cell invasion platform was used to separate and isolate a subpopulation of cells by their motility. The molecular characteristics of these high-motility cells can be further investigated, demonstrating the potential in study the mechanisms underlying heterogeneous cell function. CSCs is one important concepts used to describe a sub-fraction of tumor cells. The CSC hypothesis suggests that tumorigenic stem cells are the source of cancer. The invasion and molecular characteristics of high invasive cells observed in this study suggest a new platform to separate and isolate CSCs. As shown in Scheme 1, a small fraction of cells with higher invasion potential migrated through the paper sheets. This small subpopulation of cells showed CSC characteristics. Thus, the CiGiP cell invasion platform could be used to separate subpopulations of cells according to their motility, and to screen proteins that promote metastasis, thereby facilitating the development of therapeutic strategies for targeting CSCs. 

## 4. Conclusions

In summary, separating and isolating a subpopulation of cells by their motility was achieved by using the multi-layer CiGiP cell invasion platform. A five-layer CiGiP platform was constructed. The cell-seeding layer was sandwiched in the middle layer. After seven days of culture, only a small fraction of cells invaded through the paper sheets, showing stronger motility than cells that did not invade. The qPCR results showed elevated expression of the putative prostate CSC biomarkers, ALDH1A1, SOX2, NANOG, and OCT4, in cells with higher motility. To the best of our knowledge, this is the first separating and characterizing heterogeneous tumor cells according to their invasion potential. Moreover, the correlation between higher motility and expression of putative prostate CSC biomarkers was demonstrated. There is an urgent need to develop strategies for the prevention of metastasis. The CiGiP cell invasion platform demonstrated in this study could be used to screen proteins that promote metastasis, thereby allowing the development of therapeutic strategies for targeting CSCs.

## Figures and Tables

**Table 1:** Primers for RT-qPCR.

Gene	Primer	Sequence (5′—3′)
OCT4	F	AACCGAGTGAGAGGCAACCT
	R	ACAGAACCACACTCGGACCA
SOX 2	F	TGACCAGCTCGCAGACCTAC
	R	TCGGACTTGACCACCGAAC
NANOG	F	CGCGTTGTGATCTCCTTCTG
	R	GTCTGGTTGCTCCAGGTTGA
ALDH1A1	F	CACAGGATCAACAGAGGTTGG
	R	GTCCAAGTCGGCATCAGCTA
GAPDH	F	CCAGGTGGTCTCCTCTGACTTCAACA
	R	AGGGTCTCTCTCTTCCTCTTGTGCTC
HIF-1α	F	GCCCCAGATTCAGGATCAGA
	R	TGGGACTATTAGGCTCAGGTGAAC
HIF-2α	F	GCCACCCAGTACCAGGACTACA
	R	CCTCACAGTCATATCTGGTCAGTTCG
